# Parents’ Experiences and Perceptions of Healthcare Transition in Adolescents with Asthma: A Qualitative Study

**DOI:** 10.3390/children10091510

**Published:** 2023-09-05

**Authors:** Hyekyun Rhee, Lindsay Batek, Lynn Rew, Laurene Tumiel-Berhalter

**Affiliations:** 1 School of Nursing, University of Texas at Austin, 1710 Red River St., Austin, TX 78712, USA; ellerew@mail.utexas.edu; 2 School of Nursing, University of Rochester, 601 Elmwood Ave, Rochester, NY 14642, USA; lindsay_batek@urmc.rochester.edu; 3 Jacobs School of Medicine and Biomedical Sciences, University of Buffalo, Buffalo, NY 14203, USA; tumiel@buffalo.edu

**Keywords:** parental perceptions and experience, adolescents, asthma, healthcare transition

## Abstract

Adolescence marks a significant transition from pediatric to adult healthcare, and parents play critical roles in supporting their adolescents with chronic conditions through this process. However, little is known about parents’ experiences, perceptions, and needs during this healthcare transition. This qualitative study explores the experiences and perceptions of parents regarding the care transition of their 16–17-year-old adolescents with asthma. Nineteen mothers participated in either a focus group or individual interviews, and a content analysis was conducted on the data. Parents expressed negative emotions and various concerns about their teens’ transition readiness and asthma management. A need for early transition training for both adolescents and parents was discussed. Overall, the complexity and challenges associated with the healthcare transition of adolescents with asthma take a toll on parents, particularly when their teens are not adequately prepared to manage asthma independently. Parents need appropriate anticipatory guidance regarding the transition and skills to navigate changing roles and negotiate asthma care responsibilities with their teens. Timely interventions and support strategies for both adolescents and parents are needed to ensure the successful healthcare transition of adolescents with asthma.

## 1. Introduction

According to 2020 data [1], 9.1% of adolescents aged 15–17 years had a current asthma diagnosis in the United States (U.S.), with an even higher prevalence of 10.3% among young people aged 20–24 years. As such, asthma remains a common health concern for adolescents and young adults. Given that about 70% of adults with severe asthma had asthma before the age of 20 [2], it is critical to ensure continuity in asthma treatment and self-management from adolescence to adulthood to prevent adverse health consequences. Nonetheless, frequent interruptions in asthma care have been reported in young people undergoing healthcare transitions [3,4,5,6]. To date, transition readiness in adolescents with asthma has not been studied extensively, but available research has reported suboptimal transition readiness [4,7,8] and transition-induced emotional stress among adolescents with asthma [4,9].

Typically, transitions tend to exaggerate the vulnerability of individuals and families [10]. During the uncertain and stressful time of transition, adolescents often rely on parents for their support and guidance [8,9]. Nonetheless, parents’ roles and needs during the care transition of adolescents with chronic conditions have been overlooked in research [11]. Similarly, there is a lack of understanding about how the parents of adolescents with asthma experience the transition, perceive their teens’ readiness, and understand their own roles in this process. To address this gap, this study aims to explore the experience and perceptions of parents regarding care transitions and the readiness of their adolescents with asthma, focusing on mid-adolescents ages 16–17 years as they are approaching the critical transition time. The insights gained from this study can potentially inform the development of interventions and support strategies to facilitate a successful healthcare transition for adolescents with asthma and their parents.

## 2. Materials and Methods

### 2.1. Study Design and Study Sample

As part of a qualitative study conducted to explore the perceptions and experiences of healthcare transition among 41 mid-to-late adolescents (16–21 years-old), the parents of younger participants aged 16–17 years were also invited to participate in an interview to capture their perspectives on their teens’ care transition. Of the 20 invited parents, 19 participated in either a focus group or an individual interview, each lasting 30–50 min. Detailed methods for recruiting adolescent participants are published elsewhere [7].

### 2.2. Study Procedure and Data Collection

The study protocol received approval from the Institutional Review Boards in two academic institutions where the study originated, and data were collected. Written informed consent was obtained from all parent participants. Parent interviews were conducted either individually or in a focus group setting, depending on their availability and preference. Two focus groups were attended by 2 and 3 parents each, while 14 parents opted for individual interviews. Two female research associates (RAs) trained in qualitative research methods conducted the interviews. Focus groups were facilitated by one RA, while the second RA took notes and assisted with group logistics. Individual interviews were conducted by one RA. Semi-structured interview questions were used consistently for both focus groups and individual interviews (see Table 1 for interview questions). All interviews were audio recorded and later transcribed verbatim. To ensure accuracy, a research staff member cross-verified the final transcriptions against the recorded interviews prior to data analysis.

### 2.3. Data Analysis

For the data analysis, we conducted a qualitative content analysis [12,13] using Microsoft Word to code and analyze the interview transcriptions. In this study, we used both manifest and latent content analysis [13]. The unit of analysis was the whole interview, and the meaning units were the individual questions found in Table 1. The label of each meaning unit was referred to here as the code, whereas categories, which are the focus of qualitative analyses, were the manifest content of the text. These categories were mutually exclusive and further broken down into sub-categories or themes. Themes expressed the underlying meaning of the text, or the latent content. The Principal Investigator and a PhD-prepared research associate, who did not conduct the interviews, analyzed the interview data independently. Each developed codes, categories, and themes by labeling the responses to each interview question. Afterwards, the independently generated codes and supporting quotations were compared, and any discrepancies were reviewed and reconciled through discussion. To enhance rigor, a third member—a trained graduate research assistant—conducted separate analyses by using the coding guide developed by the first two members. Once again, any discrepancies with the first round of analyses were discussed and resolved upon mutual agreement, resulting in further refined coding. The involvement of three study team members in the data analysis and interpretation aimed to achieve the validity and reliability of the qualitative data analysis process [14].

## 3. Results

### 3.1. Sample Characteristics

This study included 19 mothers as participants. Table 2 provides an overview of the demographic characteristics of the parent participants. The sample comprised an equal representation of White and non-White parents, while only two parents identified as Hispanic.

**Figure 1:**
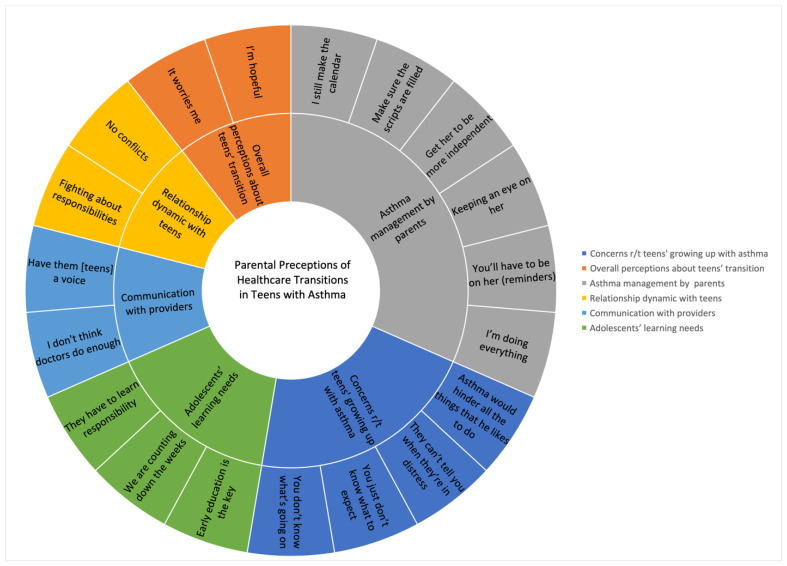
Summary of categories and themes. The inner circle of the chart depicts the six categories, or the manifest content of the text, which are also visible in the legend to the right side of the chart. These categories are further detailed by the themes provided in the outer circle of the chart. The themes reflecting the underlying meaning, or latent content, of the text are direct quotations from parent interviews and indicate the perceptions that parents shared regarding their teens’ healthcare transition.

Data were organized into six categories pertinent to healthcare transition in teens with asthma, under which several subcategories or themes emerged representing parents’ perspectives and experiences. The themes were labeled using phrases from direct quotes of parents. The direct quotes supporting the themes are italicized below. Figure 1 illustrates six categories and corresponding themes. Below are the descriptions of each category and associated themes.

### 3.2. Concerns Related to Teens Growing up with Asthma

In general, parents experience negative emotions when responding to their teens’ symptoms, including stress, frustration, helplessness, worry, and fear. These reactions are often related to their lack of awareness about symptom situations, symptoms becoming unpredictable or worsening with age, and their teens’ insufficient preparation to recognize and manage symptoms and triggers.

#### 3.2.1. You Don’t Know What’s Going On

Parents expressed their frustration over not knowing about situations that might be responsible for their teens’ symptoms. The fact that teens do not always share their whereabouts with their parents leaves them bewildered and fearful when their teens suffer from symptoms. As a result, parents often feel unsure of how to intervene effectively to reduce or remove triggers for these symptoms.

*[I]t was very frightening when you don’t know what’s going on…And you’re wondering what the heck is going on…So we were trying to pinpoint it. … That’s the one thing is that you just don’t know.*

Adolescents’ increasing independence triggered negative emotional reactions in some parents, as they feared not being informed about their teens’ asthma condition until it was too late.

*To be honest with you, [my teen] is more responsible about her health than I am about her own health, which scares me…she’s more about it [asthma] than I am. I don’t…I don’t think about that stuff until it’s too late…it’s still that nerve-wrecking thing.*

Some parents felt helpless and concerned about their limited ability to help their teens, as they were unsure about the exact cause of the symptoms. This uncertainty left them worried that healthcare providers might criticize them as being ‘unfit mothers’.

*If you don’t do stuff right, doctors will think you are very—um—what they call it? Um, unfit mother. And that’ll really piss you off.*

#### 3.2.2. You Just Don’t Know What to Expect

Some parents experience emotional stress due to the unpredictable nature of asthma symptoms.

*Sometimes it’s better, sometimes it’s not. Is there a possibility of maybe it getting worse? You know, you just don’t know what to expect. You don’t know if she’s gonna have a good week or it’s never gonna happen again or when it’s going to. So it’s stressful.*

While some parents noted that their teens’ asthma had become less severe compared to when they were younger, many others remained concerned about the lack of improvement or even worsening of asthma symptoms with age. This constant source of stress or fear was particularly prominent as parents felt a loss of control over their teens’ lives as they became more independent.

#### 3.2.3. They Can’t Tell You When They’re in Distress

A few parents expressed concerns about their teens’ inability to properly recognize or manage triggers or symptoms when the parents were unavailable to assist them.

*[I]t could be somewhat stressful because they can’t tell you when they’re in distress.*

*She just-like very tight and really couldn’t breathe… not knowing why she’s having difficulty performing her daily activities.*

A parent also mentioned that her teen refused to take medication, which was administered through a nebulizer.

#### 3.2.4. Asthma Would Hinder All the Things That He Likes to Do

Some parents raised a concern about asthma potentially preventing their teens from participating in certain activities in the future, such as playing basketball, or possibly passing the condition to their offspring.

*I guess maybe the biggest thing is if it’s genetic or if she has children maybe someday.*

### 3.3. Overall Perceptions about Teens’ Healthcare Transition

#### 3.3.1. I’m Hopeful

When asked about their thoughts regarding their teens becoming adults with asthma, some parents denied having any concerns and remained hopeful that the symptoms would be less severe or that their teens would eventually ‘grow out of’ asthma. For some parents, the hope of better-controlled asthma was based on the assumption that their teens would become less active over time, leading to fewer symptoms.

*[I am] hopefully when she goes to college next year…it might not be as bad I’m hoping, because she won’t be in as many sports. She’s very active right now.*

Others remained optimistic, believing that their teens would continue to be responsible in taking care of asthma as adults.

*I don’t really worry about it because she’s pretty responsible and I know that she’ll always have her inhaler with her if she needs it.*

*[S]ince she’s had it for so long we know what the triggers are, and so if she feels she’s gonna be in a situation where she would need rescue, she almost kinda takes it like proactively.*

While some parents were not entirely convinced of their teens’ ability to manage asthma independently, they found hope in noticing recent improvements in their teens’ trigger perception or medication adherence. Despite denying any problems with their teens managing asthma independently, a few parents admitted that their teens would still need help during asthma flare-ups.

*He was pretty good about taking medications, but it never seemed to help when he was sick. And he didn’t really have any problems except when he was sick.*

#### 3.3.2. It Worries Me

Despite few parents expressing positive sentiments about the upcoming care transition for their teens, the majority shared concerns that their teens were inadequately prepared. Many believed that their teens lacked organization and had insufficient knowledge and skills to self-manage asthma independently. Overall, the parents lacked confidence in their teens’ capability to recognize and manage symptoms and triggers properly and to reliably take medication, as illustrated in the following statement.

*I don’t know that he’s going to pay attention to it as much as I did. I don’t know if it will bother him that much, though. I don’t know if he’s really going to care. I don’t know. And even the way he is now, he doesn’t want to take…he really just doesn’t want to have to [take medication] anymore. Yeah, I don’t know if he’s going to follow through… will he follow their directions if there’s nobody there telling him what to do? I don’t know. I don’t think so.*

Parents were not only concerned about their teens’ inability to control symptoms but also worried about negative emotions their teens might experience when responding to acute symptoms, such as feeling scared, worried, and embarrassed. Some parents even admitted that they themselves were not fully ready to see their teens launch into independent adulthood.

*I’m nervous for college…I don’t know if I’m ready for that. I haven’t even gotten to that. I’m thinking about it, but in her mind, I’m moving with her [to college].*

In addition, some parents had doubts about their teens’ transition readiness due to their teens’ reckless engagement in risk behaviors that triggered symptoms, such as vaping.

*I’ve been worried about the vaping thing…we found out she was doing that this past year… last year she had an asthma attack, so we had to call the ambulance, and it turns out that was because of that.*

Switching the teens’ providers was also perceived as a source of stress for many parents. Their concerns revolved around three unknown qualifications of ‘adult doctors’: first, the proficiency to manage symptoms (“*make things as urgent as the pediatrician does*”); second, attentiveness (“*they would dismiss a lot of what they need to pay attention to*”); and third, continuity of care (“*does everything follow or does this new PCP try to re-diagnose*”).

Despite the expressed concerns and doubts about their teens’ care transition, the majority denied having any conversation with their teens’ providers about it. Only one parent affirmed that a topic of care transition was brought up during the appointment, while three parents responded that they planned to make an appointment with their teens’ providers to discuss care transition.

### 3.4. Asthma Management by Parents

Overall, the parents acknowledged that their teens had become more prepared for managing asthma symptoms in recent years, mainly judging by their carrying and using rescue medication. Only two parents mentioned the teens’ regular use of controller medication in the context of symptom prevention. The responsibility of managing their prescriptions was not routinely carried out by the teens, with only one parent stating that her teen would contact the pharmacy for medication refills. Monitoring symptoms or triggers was seldom brought up. Several parents did notice their teens’ growing confidence in communicating and advocating for their asthma-related needs. Although teens are becoming more independent in managing their asthma, many parents still play an active role in various aspects of their child’s asthma management to varying degrees.

#### 3.4.1. I’m Doing Everything

Some parents continue to be over-involved in the teens’ asthma management. They justified their overinvolvement to compensate for their teens’ neglect of the responsibility for asthma care. Consequently, these parents took charge of almost every aspect of asthma management, although they recognized the challenges of maintaining control as their teens grew older.

#### 3.4.2. You’ll Have to Be on Her (Reminders)

Most parents emphasized that their constant reminders were crucial to ensure that their teens kept their rescue medication with them at all times and adhered to control medication as scheduled.

#### 3.4.3. Keeping an Eye on Her

Parents constantly monitor their teens’ symptoms and intervene whenever necessary to manage them. Often, parents notice changes in symptoms before their teens do, enabling them to provide timely management and effectively prevent exacerbations.

#### 3.4.4. Try to Get Her to Be More Independent

Most parents take on multiple roles as motivators, cheerleaders, coaches, teachers, and role models for their teens. By doing so, they aim to prepare their teens to become more independent and responsible in managing their asthma.

*I would say maybe just give him certain advice about, you know, how to take care of himself or certain things that he needs to do. To be a little more independent, you know, to not depend on me to do it.*

#### 3.4.5. Make Sure the Scripts Are Filled

The responsibility of managing prescriptions primarily falls on the parents. As not all teens communicate when their medication needs refilling, parents often monitor their teens’ medication usage to determine when refills are necessary, ensuring the medication is available when needed.

#### 3.4.6. I Still Make the Calendar

Parents take an active role in scheduling clinical appointments for their teens and accompany them, often communicating with the healthcare provider on their behalf. They believe that teens may not have the experience or knowledge to ask the right questions and engage in effective communication during medical appointments. So, these parents expect to continue accompanying their teens to clinical appointments even after they become adults to ensure the best possible care.

### 3.5. Relationship Dynamics with Adolescents

#### 3.5.1. No Conflicts

When asked about any difficulties in their relationships with their teens around asthma management, only four parents denied any conflicts who were content with the ways of asthma being managed by their teens.

#### 3.5.2. Fighting about Responsibilities

The majority of parents reported experiencing some degree of conflict with their teens. The primary cause of conflict was issues around medication adherence. Some parents expressed frustration about their teen’s carelessness (e.g., misplacing inhaler) or refusal to take medication. Teens would neglect to take medication or argue against it, stating “*I might not need it*” or even denying they have asthma at all. In some cases, teens refused medication due to concerns about its side effects or perceived ineffectiveness, as described in the following statements.

*It’s the struggle with the medications, really that she…what works, what doesn’t work, what she feels. “Why do I have to keep taking this if I feel like this doesn’t take effect?” …I think that’s the biggest struggle me and her have, it’s just the steroid cause she doesn’t like the way it makes her feel.*

A few parents mentioned that the conflicts had improved with their teens becoming more responsible for taking medication.

### 3.6. Communication with Providers

When asked about the primary communicator during the appointments, there were mixed responses, with some parents being the primary communicators while others mentioned their teens being the sole communicator. Many teens were reported to take an active role in communicating with the provider, but parents’ encouragement or prompting was often necessary. Some parents assumed the role of spokesperson for their teens who were reluctant to speak with the provider due to various reasons, such as feeling uncomfortable or intimidated, dissatisfaction with the provider, or lacking communication skills. Two themes emerged describing parental perspectives in relation to teens’ communication with healthcare providers.

#### 3.6.1. Have Them [Teens] a Voice

Parents invariably expressed the desire for their teens to become more confident and comfortable in communicating their needs and to “*break out of the shell*” in preparation to become independent adults.

*When they get to that point, the understanding that’s who they have to rely on, that when they get to the age we have to step back. That we won’t be able to do any of this stuff anymore. I think that, that’s when I think they’ll eventually breakout of the shell. It’ll be a while but I think it’ll be a little while.*

Many parents emphasized the importance of early communication training for their teens to “*have them a voice*”, so that parents would be available to provide support if needed.

*My daughter’s 17 and heading off to college… You know so like that transition should actually start a little bit sooner… Like to advocate more for themselves and speak more for themselves maybe, at maybe a little bit earlier of an age. Still have mom and dad there… let them [providers] talk to more to the child rather than the parent.*

Even so, some parents were adamant in their belief that it was the necessity for them to be part of the conversation because their teens tended to ‘*deny*’ or ‘*downplay things*’, and sometimes omitted important information or questions during appointments.

#### 3.6.2. Don’t Think Doctors Do Enough

Some parents expressed a desire for the providers to make more efforts to engage the teens in conversations, as illustrated in the following statement.

*I don’t think the doctors do enough of that, of asking [the teen]…cause we’re not them…we don’t know their asthma’s feeling…We can’t tell you how they actually feel…If they ask the kids…I think it would help them a little bit more to be able to advocate for themselves and as they get older it won’t be so hard.*

At the same time, parents also described the need for the providers to improve their communication skills to effectively engage teens in meaningful conversations, enabling them to have a more active role in their healthcare decisions.

*I feel like the doctor needs to be able to ask things in a non-threatening way to a teen or in a-in vocabulary that a teen can relate to. She [Provider] didn’t explain to her [my teen] why the things like “this is what’s going on and this is why we’re giving you this medicine. And this is why we’re giving you this medicine, and this is what this is going to do for you…and how this is going to make you feel…” And I think that it needs to be broken down for kids; for the doctor to talk to the teen, and not to the parent.*

### 3.7. Adolescents’ Learning Needs

#### 3.7.1. They [Teens] Have to Learn Responsibility

Parents expressed their lack of confidence in their teen’s capacity to self-manage asthma, believing that their teens still need reminders to take medication and avoid triggers. Most parents strongly desired for their teens to take greater ownership of caring for their asthma until becoming fully in charge of their health.

#### 3.7.2. We Are Counting Down

Parents recognized the urgency of the impending care transition and their teens’ immediate need to learn how to manage asthma independently with confidence and competence.

*We’re counting down the weeks of all the independent living skills he has to learn.*

#### 3.7.3. Early Education Is the Key

Regarding the timing of education or training for care transition, parents wanted it to take place as early as 12 or 13 years of age so that they could be available to provide support if needed. This was particularly relevant because some teens might leave home for college as early as 17.

## 4. Discussion

To the best of our knowledge, this is the first study investigating the perceptions and experiences of parents with 16–17-year-old adolescents with asthma approaching adulthood. Overall, we found that parents commonly experience negative emotions in response to their teens’ asthma and becoming adults. As their teens become more independent and less likely to share information about their condition, parents feel frustrated and uneasy about not knowing about their teens’ asthma condition and when and how to help during symptom experiences. These changing dynamics may lead parents to grieve the loss of their familiar and prominent roles as key players in managing their child’s chronic health condition during the transition, as reported in previous studies [15,16,17]. Similar to the parents of adolescents with more complex chronic conditions [15,16], our parents fear that their teens will not be able to recognize or manage their symptoms properly when they are not around. Furthermore, they expressed concerns about the unpredictability of asthma symptoms and the possibility of symptoms worsening as their adolescents grow older. They also worried about how asthma might limit future opportunities for their teens and the potential intergenerational transmission of asthma affecting their teens’ future offspring.

Parents’ thoughts about their teens’ impending healthcare transition can be summarized in three ways. First, the most common response was that, similar to their teens [9], many parents simply have not thought about the transition, despite the aforementioned negative feelings. Second, few parents held a positive outlook based on assumptions that their teens would ‘grow out of asthma’ or become better at managing asthma as they get older. Because these assumptions often turn out to be false, disappointment and frustration can result when reality proves otherwise. Third, the most prevailing sentiment among most parents who have considered their teens’ approaching care transition was worry and uncertainty. This concern primarily stems from their teens’ limited preparedness to manage asthma independently. Unlike their teens who expressed high confidence in managing asthma independently [9], parents were worried about their teens’ lack of knowledge, skills, or emotional maturity to self-manage asthma, and their lifestyle choices that could potentially aggravate the condition.

Additionally, switching providers associated with care transition was also found to be a cause of stress for many parents related to losing connection to the pediatric providers, and the uncertainty regarding the competency and continuity of care from adult providers, as reported in other studies [11,18,19,20,21]. An opportunity to meet with the adult provider or visit the adult clinic with their teens as part of the transition process could potentially alleviate such stress in parents, as suggested by several studies [22,23] However, to our surprise, the topic of care transition was rarely brought up during their teens’ clinical appointments, corroborating their teens’ report [9] and an earlier study [24]. Parents highly value providers’ efforts to prepare them by notifying them in a timely manner about the timing and process of the approaching transition, and by involving them in the planning [22]. The perceived inattention of providers to this aspect raises a concern. Furthermore, the providers’ failure to address the transition-related needs in both parents and adolescents may be a contributor to widespread deficiencies in transition readiness in young people with chronic conditions [25,26].

Some parents perceived that their teens were prepared to self-manage asthma solely because they improved in carrying and using rescue medication, with little attention to other aspects of asthma management such as symptom monitoring and prevention, or trigger monitoring and management. This narrow view of asthma self-management may lead parents to overestimate their teens’ capability to manage asthma independently. Nonetheless, consistent with the literature [11], parents continue to play critical roles in their teens’ asthma management, particularly in (re)filling and reminding about medication, monitoring asthma conditions, managing clinical appointments, and providing moral and informational support for their teens as they become independent managers of their asthma. Parents perceived their continual involvement and support as critical and necessary to protect their teens from any serious consequences of asthma until the teens become fully ready to manage their asthma responsibly. This finding aligns with an earlier study that reported parents’ general hesitancy to release the responsibilities of disease management to their teens [27].

Conflicts arise when the parents’ desire to stay involved collides with teens’ desire for a “hands-off” approach [9]. Interestingly, contrary to most teens denying any conflicts with their parents in our previous report [9], varying degrees of disputes were reported by their parents regarding asthma management. As causes for the conflicts, parents cited teens’ neglect or refusal to take medication or denial of having asthma, which have been related to declining medication adherence as adolescence progresses [28,29,30]. Therefore, parental involvement seems essential to ensure their child’s adequate disease management, although it is not always appreciated by teens [31,32,33]. This underscores the need for proactive guidance for parents to strategically support and negotiate responsibilities of asthma management with their teens, to minimize conflicts and to promote better asthma care coordination within the family.

Many parents still felt the need to be present during healthcare appointments to ensure that critical information about their teens’ asthma is communicated to and with providers. This benefit was also recognized by their teens, although it could impede teens’ direct communication with providers [9]. The parents believed that their teens could benefit from training to gain more confidence and competence in communicating with providers. They urge providers to make concerted efforts to skillfully engage teens in conversation in a non-threatening manner. Positive relationships and better communication between adolescents and their providers have been associated with improved adherence to self-management [34,35,36,37,38,39], and greater motivation to take medication independently in adolescents with asthma [40,41]. Therefore, providers’ efforts to establish and maintain positive and trusting relationships with adolescents through good communication skills (e.g., listening and empathizing) is important in enhancing adolescents’ self-management that is essential to a successful healthcare transition.

This study has several limitations. First, caution is needed in generalizing the findings to parents of younger adolescents aged <16 years, or fathers, as only mothers participated in this study. Second, meaningful differences between minority and White parents were not found, possibly due to the small sample size. However, some experiences may be unique to specific race or social subgroups due to differences in parenting styles, expectations, and socioecological contexts. Further research is needed to explore these unique circumstances and how they contribute to different parent experiences and needs for their transitioning teens, which can guide the development of more tailored interventions to support parents. Finally, it is important to note that our findings are solely based on parents’ perspectives and may not fully represent how the healthcare transition of teens with asthma is managed in clinical practices. Further research involving both parents and the healthcare providers can provide a more comprehensive understanding of the transition care practices in healthcare facilities.

## 5. Conclusions

This study demonstrates that the parents of adolescents with asthma experience a wide range of vulnerabilities due to multiple challenges associated with their teens’ healthcare transition. In general, parents are concerned and frustrated about the gradual loss of control over their teens’ asthma management and uncertainty about the disease trajectory and its impact on their child’s future. As such, it is important to identify and address parent concerns by providing appropriate anticipatory guidance, emotional support, and information. Parents question their teens’ readiness to properly manage asthma independently and remain actively involved in various aspects of disease management for their child. This highlights the need to support parents and teens during this critical transition period, offering education and training to empower teens in independent asthma management while ensuring parents’ confidence in their teens’ capabilities. Early intervention appears critical not only for adolescents to improve their competence for asthma self-management, but also for parents to establish a strategic partnership with their teens. Providing parents with skills to navigate their changing roles, through gradually negotiating and delegating the responsibilities of asthma management to adolescents and offering timely support and oversight, can prepare them for their teens’ transition. The perceived inattention of providers to adolescents’ healthcare transition, whether real or not, highlights the need for a more targeted and structured approach from providers in assessing and addressing transition readiness in 16–17-year-old adolescents with asthma. This study calls for attention to be paid to parents’ concerns and roles, while recognizing them as relevant and important partners in adolescents’ healthcare transition and offering interventions to support both parents and teens throughout the process.

## Figures and Tables

**Table 1:** Parent interview questions.

	Interview Questions
Overall experiences and perspectives	▪ What has it been like for you to parent a child with asthma?
	▪ Have you thought about your child becoming an adult with asthma? What worries/excites you most when you think about them being a grown-up with asthma?
	▪ Has anyone in your doctor’s office discussed with you and your child about how they become independent in asthma care and switch to an adult practice? If yes, what specifically did your doctor tell you and your child about transitioning to adult care?
	▪ What are your concerns in relation to the transition?
Transition readiness and needs	▪ What role do you play currently in your child’s asthma management?
	▪ What role does your child play in their own asthma care? ▪ What are the conflicts that arise with your child due to their asthma? ▪ How much do you communicate directly with your child’s provider on your child’s behalf during an appointment?
	▪ How confident are you that your child could carry out the tasks of asthma management on their own when they are no longer a teenager?

**Table 2:** Sample characteristics.

Characteristics/ Variables	M (SD) Range	N	%
Age (years)	45.87 (5.27) 34–56		
Relationship to teens			
Mother		19	100
Parent relationship status			
Married		8	42.1
Single but with a partner		7	36.9
Never married		1	5.2
Missing		3	15.8
Race			
White		8	42.1
Minority (African American/Hispanic)		8	42.1
Missing		3	15.8
Household income (annual)			
<USD 30,000		7	36.9
≥USD 30,000		9	47.3
Missing		3	15.8

## Data Availability

The data presented in this study are available on request from the corresponding author. The data are not publicly available due to privacy concerns.
